# Brain Map of Intrinsic Functional Flexibility in Anesthetized Monkeys and Awake Humans

**DOI:** 10.3389/fnins.2019.00174

**Published:** 2019-02-28

**Authors:** Dazhi Yin, Zhao Zhang, Zhiwei Wang, Kristina Zeljic, Qian Lv, Danchao Cai, Yingwei Wang, Zheng Wang

**Affiliations:** ^1^Institute of Neuroscience, State Key Laboratory of Neuroscience, Key Laboratory of Primate Neurobiology, CAS Center for Excellence in Brain Science and Intelligence Technology, Shanghai Institute for Biological Sciences, Chinese Academy of Sciences, Shanghai, China; ^2^Department of Anesthesiology, Huashan Hospital, Fudan University, Shanghai, China; ^3^University of Chinese Academy of Sciences, Beijing, China

**Keywords:** dynamic brain organization, intrinsic functional flexibility, evolution, monkey, anesthesia, resting-state fMRI

## Abstract

Emerging neuroimaging studies emphasize the dynamic organization of spontaneous brain activity in both human and non-human primates, even under anesthesia. In a recent study, we were able to characterize the heterogeneous architecture of intrinsic functional flexibility in the awake, resting human brain using time-resolved analysis and a probabilistic model. However, it is unknown whether this organizational principle is preserved in the anesthetized monkey brain, and how anesthesia affects dynamic and static measurements of spontaneous brain activity. To investigate these issues, we collected resting-state functional magnetic resonance imaging (fMRI) datasets from 178 awake humans and 11 anesthetized monkeys (all healthy). Our recently established method, a complexity measurement (i.e., Shannon entropy) of dynamic functional connectivity patterns of each brain region, was used to map the intrinsic functional flexibility across the cerebral cortex. To further explore the potential effects of anesthesia, we performed time series analysis and correlation analysis between dynamic and static measurements within awake human and anesthetized monkey brains, respectively. We observed a heterogeneous profile of intrinsic functional flexibility in the anesthetized monkey brain, which showed some similarities to that of awake humans (*r* = 0.30, *p* = 0.007). However, we found that brain activity in anesthetized monkeys generally shifted toward random fluctuations. Moreover, there is a negative correlation between nodal entropy for the distribution of dynamic functional connectivity patterns and static functional connectivity strength in anesthetized monkeys, but not in awake humans. Our findings indicate that the heterogeneous architecture of intrinsic functional flexibility across cortex probably reflects an evolutionarily conserved aspect of functional brain organization, which persists across levels of cognitive processing (states of consciousness). The coupling between nodal entropy for the distribution of dynamic functional connectivity patterns and static functional connectivity strength may serve as a potential signature of anesthesia. This study not only offers fresh insight into the evolution of brain functional architecture, but also advances our understanding of the dynamics of spontaneous brain activity.

## Introduction

A fundamental goal of comparative neuroscience is to determine conservation and evolution-driven changes in functional brain organization between species. Functional magnetic resonance imaging (fMRI), a non-invasive technique, has been utilized to identify functionally homologous or unique areas across primate species based on specific experimental tasks (Nakahara et al., 2002; Vanduffel et al., 2002; Wang et al., 2015). This technique has the advantage of providing a direct cross-species comparison using a common physiological measurement, i.e., blood oxygen level-dependent (BOLD) signal. Owing to ease of implementation and the robustness of findings, resting-state fMRI, simply a period of recording of BOLD signal in the absence of any explicit tasks, has become an attractive tool for studying large-scale brain functional organization (Zhang and Raichle, 2010; Power et al., 2014). Resting-state functional connectivity (FC) is typically used to index the interregional coherence in spontaneous low-frequency fluctuations (e.g., 0.01∼0.1 Hz) of BOLD signals (Biswal et al., 1995; Fox and Raichle, 2007). Through this approach, many functional brain networks such as the sensorimotor and default mode networks have been identified in both human and non-human primates (Vincent et al., 2007; Margulies et al., 2009; Hutchison et al., 2011; Hutchison and Everling, 2012; Mantini et al., 2013; Miranda-Dominguez et al., 2014; Neubert et al., 2014). In particular, the patterns of resting-state FC have showed some similarities across species and appear to transcend levels of consciousness, being present under anesthesia, see Raichle (2015) for a review. However, conventional resting-state FC is frequently evaluated in a time-averaged sense, under the potential assumption of stationary functional organization. Moreover, the origins and functional significance of resting-state FC patterns remain to be further understood.

Recently, emerging studies have emphasized the dynamic organization of brain function, suggesting that understanding brain function and dysfunction requires an integrated framework linking brain connectivity and brain dynamics (Deco et al., 2011; Liu and Duyn, 2013; Calhoun et al., 2014; Kopell et al., 2014; Braun et al., 2015; Christoff et al., 2016). Therefore, dynamic FC analysis, e.g., taking into account the temporal fluctuations of FC in different time windows of BOLD signals, has been proposed to characterize spontaneous brain activity (Chang and Glover, 2010; Zalesky et al., 2014; de Pasquale et al., 2015; Karahanoglu and Van De Ville, 2015; Chen et al., 2016). Although a number of challenges in techniques and interpretation remain, time-resolved analysis allows researchers to extract more in-depth information about brain function than static FC analysis (Jia et al., 2014; Keilholz et al., 2017; Liegeois et al., 2017; Preti et al., 2017).

Developing new analytic tools to describe spatiotemporal characteristics of resting-state FC patterns across species and states may provide deeper insight into functional organization of spontaneous brain activity. Based on dynamic FC analysis and clustering method, many discrete, reproducible functional states over the time of scan have been identified in both humans and monkeys (Allen et al., 2014; Barttfeld et al., 2015), see Hutchison et al. (2013a); Calhoun et al. (2014) for reviews. In contrast, our recent work (Yin et al., 2016) focused on quantifying the flexibility of the connectivity pattern for each brain region over time, using a complexity measurement (i.e., Shannon entropy) and probabilistic model. Consistent with task-induced functional reconfigurations (Cole et al., 2013; Braun et al., 2015), we revealed the heterogeneous organization of functional flexibility in the resting human brain. However, it is unknown whether this organizational principle is preserved in the anesthetized monkey brain, and how it corresponds with the human brain during wakeful rest. Although the spatiotemporal dynamics of brain activity have been demonstrated in the anesthetic state (Hutchison et al., 2013b; Barttfeld et al., 2015; Zhang et al., 2018), few studies pay attention to the cross-species correspondence of brain-wide dynamic organizational structure. Moreover, how anesthesia affects dynamic and static measurements of spontaneous brain activity still needs to be clarified.

To investigate these issues, we collected resting-state fMRI datasets from 178 awake humans and 11 anesthetized monkeys (healthy subjects). Our recently established method (Yin et al., 2016), a complexity measurement (i.e., Shannon entropy) of dynamic FC patterns of each brain region, was used to map the intrinsic functional flexibility across the cerebral cortex. Brain regions with high entropy for the distribution of dynamic FC patterns indicate high functional flexibility, and vice versa. For comparison, we conducted another complexity measurement based on distribution of correlation values, which reflects the functional complexity of a system (Zhao et al., 2010; Zamora-Lopez et al., 2016). A temporal variability analysis as a measure of functional flexibility was also carried out (Mueller et al., 2013; Zhang et al., 2016). To further explore the potential effects of anesthesia on the functional organization of spontaneous brain activity, we performed time series analysis and correlation analysis between dynamic and static measurements within awake human and anesthetized monkey brains, respectively. We hypothesized that the heterogeneous organization of intrinsic functional flexibility in the awake human brain persists in the anesthetized monkey brain.

## Materials and Methods

### Participants

All experimental procedures for non-human primate research in this study were approved by the Institutional Animal Care and Use Committee at the Institute of Neuroscience and the Biomedical Research Ethics Committee, Shanghai Institutes for Biological Sciences, Chinese Academy of Sciences, and conformed to National Institutes of Health guidelines for the humane care and use of laboratory animals.

We recruited 11 wild-type monkeys (age 4.68 ± 0.46 years; weight 3.97 ± 1.36 kg; 7 female). In addition, 178 healthy human subjects (age 14.4 ± 3.6 years; 44 female; 35 subjects with eyes closed and 143 subjects with eyes open) were collected from the Autism Brain Imaging Data Exchange (ABIDE)^1^. We screened the human data based on demographic and diagnostic information provided in the ABIDE database (Di Martino et al., 2014). The inclusion criteria in the present study are briefly described as follows: (1) right-handedness, (2) age between 7 and 22 years, (3) a full-scale IQ score greater than 70; and time resolution of fMRI data equal to 2 s (datasets from four sites fit this criterion, including NYU, YALE, TRINITY, and UM).

### Monkey Data Acquisition

Magnetic resonance imaging images of monkeys were acquired at the Institute of Neuroscience on a 3T whole-body scanner (Trio; Siemens Healthcare, Erlangen, Germany) running with an enhanced gradient coil insert (AC88; 80 mT/m maximum gradient strength, 800 mT/m/s maximum slew rate). A custom-built 8-channel phased-array transceiver coil was used for animal imaging sessions. Whole-brain resting-state fMRI data were collected using a gradient-echo echo-planar imaging (EPI) sequence (TR = 2000 ms; TE = 29 ms; flip angle = 77°; slices = 32; matrix = 64 × 64; field of view = 96 mm × 96 mm; 1.5 mm × 1.5 mm in plane resolution; slice thickness = 2.5 mm; GRAPPA factor = 2). For each session, 5–10 runs were acquired and each run consisted of 200 functional volumes. A pair of gradient echo images (echo time: 4.22 and 6.68 ms) with the same orientation and resolution as EPI images were acquired to generate a field map for distortion correction of EPI images. High-resolution T1-weighted anatomical images were acquired using a MPRAGE sequence (TR = 2500 ms; TE = 3.12 ms; inversion time = 1100 ms; flip angle = 9°; acquisition voxel size = 0.5 mm × 0.5 mm × 0.5 mm; 144 sagittal slices). Six whole-brain anatomical volumes were acquired and further averaged for better brain segmentation.

For MRI scanning, animals were prepared and maintained in a stable brain state under light anesthesia. The animal preparation procedure was conducted in a manner similar to our previous work (Wang et al., 2013; Lv et al., 2016). Induction of anesthesia was achieved by intramuscular injection with ketamine (10 mg/kg, Gutian Pharma Co., Ltd., China) before MRI scanning sessions, supplemented with atropine sulfate (0.05 mg/kg, Shanghai Harvest Pharma Co., Ltd., China) to decrease bronchial and salivary secretions. After intubation, animals were ventilated with a mixture of isoflurane (2–2.5%, Lunan Pharma Co., Ltd., China) and oxygen via either a standard ventilator (CWE, Inc., Ardmore, PA, United States) outside the scanner room or an MRI-compatible ventilator (CWE Inc., Weston, WI, United States) inside the scanner room. Macaques were maintained with intermittent positive-pressure ventilation to ensure a constant respiration rate (25–35 breaths/min). The concentration of isoflurane was adjusted based on continuously monitored vital signs, including blood oxygenation, electrocardiogram (ECG), rectal temperature (Small Animal Instruments, Inc., Stony Brook, NY, United States), respiration rate and end-tidal CO_2_ (Smiths Medical ASD Inc., Dublin, OH, United States). Oxygen saturation was kept over 95% and body temperature was kept constant using a heated water blanket (Gaymar Industries Inc., Orchard Park, NY, United States). Lactated Ringer’s solution was given with a maximum rate of 10 ml/kg/h during the anesthesia process (Logothetis et al., 1999). We removed the runs that showed erratic vital signs, image artifacts, as well as burst suppression according to the recordings of MRI-compatible electroencephalograph (Brain Products GmbH, Gilching, Germany) during functional data acquisition. In total, 99 runs were left for the final analyses. We treated each run independently following previous studies (Barttfeld et al., 2015; Lv et al., 2016).

### Human Data Acquisition

Human MRI data were acquired from multiple sites with different parameters of pulse sequences (see text footnote 1). In the present study, one of the inclusion criteria was a time resolution of fMRI data equal to 2 s, to match the monkey data. In addition, we kept the same number of time points (i.e., 150 brain volumes) used across different sites.

### Preprocessing of Monkey and Human fMRI Data

Functional brain images of monkey and human were preprocessed using the same steps, including slice timing correction, motion correction, coregistration with individual T1-weighted image, normalization to the corresponding standard space, resampling and spatial smoothing, regression of nuisance signals, removal of linear drift, and temporal filtering.

Specifically, the preprocessing of the monkey data were done using the SPM 8.0 toolbox^2^ and the FMRIB Software Library toolbox (FSL^3^). The first 10 volumes were discarded. The field map images of each participant were then applied to compensate for the geometric distortion of EPI images caused by magnetic field inhomogeneity using FSL FUGUE. After slice timing correction and motion correction, the corrected images were normalized to standard space of the monkey F99 atlas^4^ using an optimum 12-parameter affine transformation and non-linear deformations, and then resampled to 2-mm cubic voxels and spatially smoothed with a 4 mm full-width at half-maximum isotropic Gaussian kernel. Six head motion parameters, ventricle, and white matter signals were removed from the smoothed volumes using linear regression. Linear drift of the volumes was removed and temporal filtering (0.0025–0.05 Hz) (Vincent et al., 2007; Barttfeld et al., 2015; Lv et al., 2016) was performed.

The preprocessing of human data was performed by the Preprocessed Connectomes Project (PCP^5^) using the Data Processing Assistant for Resting-State fMRI (DPARSF) Toolbox (Yan and Zang, 2010). Preprocessing steps included slice timing correction, motion correction, spatial normalization into MNI space, resampled to 3 mm × 3 mm × 3 mm voxels and smoothing with a Gaussian kernel (full-width at half-maximum = 6 mm). Friston-24 parameters of head motion, white matter and ventricle signals were regressed out, followed by linear drift correction and temporal filtering (0.01–0.1 Hz). For more details, readers may refer to the description in the PCP^6^.

### Static and Dynamic FC Analysis

For both preprocessed monkey and human datasets, we first divided the brain into different areas. For a direct cross-species comparison, here we adopted a regional map (RM) parcellation (82 cortical regions) for both monkeys and humans (Table 1), which is based on a combination of microstructural, functional, and topographic features (Kotter and Wanke, 2005; Bezgin et al., 2012; Reid et al., 2016). This parcellation has the same terminology for the monkey and human brains, but the topographic assignments originate from the monkey cortex. Pearson’s correlation coefficients between the mean time courses of any pair of regions over the whole scan were then calculated to represent static FC, resulting in an 82 × 82 connectivity matrix. Finally, Fisher’s Z-transformation was applied to the connectivity matrix so that their distributions could better satisfy normality.

**Table 1:** Brain parcellation of regional map.

Label	Abbreviation	Full name
1/2	TCpol	Temporal polar cortex
3/4	Amyg	Amygdala
5/6	PHC	Parahippocampal cortex
7/8	TCi	Inferior temporal cortex
9/10	TCv	Ventral temporal cortex
11/12	HC	Hippocampal cortex
13/14	TCc	Central temporal cortex
15/16	TCs	Superior temporal cortex
17/18	VACv	Anterior visual cortex, ventral part
19/20	V1	Primary visual cortex
21/22	PFCoi	Orbital inferior prefrontal cortex
23/24	V2	Secondary visual cortex
25/26	PFCom	Orbitomedial prefrontal cortex
27/28	Ia	Anterior insula
29/30	Ip	Posterior insula
31/32	CCs	Subgenual cingulate cortex
33/34	PMCvl	Ventrolateral premotor cortex
35/36	CCp	Posterior cingulate cortex
37/38	CCr	Retrosplenial cingulate cortex
39/40	G	Gustatory cortex
41/42	PFCol	Orbitolateral prefrontal cortex
43/44	A2	Secondary auditory cortex
45/46	PFCvl	Ventrolateral prefrontal cortex
47/48	A1	Primary auditory cortex
49/50	VACd	Anterior visual cortex, dorsal part
51/52	S2	Secondary somatosensory cortex
53/54	PFCpol	Prefrontal pole cortex
55/56	S1	Primary somatosensory cortex
57/58	PFCm	Medial prefrontal cortex
59/60	PCm	Medial parietal cortex
61/62	M1	Primary motor cortex
63/64	FEF	Frontal eye field
65/66	CCa	Anterior cingulate cortex
67/68	PFCcl	Centrolateral prefrontal cortex
69/70	PCip	Intraparietal cortex
71/72	PCi	Inferior parietal cortex
73/74	PCs	Superior parietal cortex
75/76	PFCdm	Dorsomedial prefrontal cortex
77/78	PFCdl	Dorsolateral prefrontal cortex
79/80	PMCdl	Dorsolateral premotor cortex
81/82	PMCm	Medial premotor cortex

*Odd numbers denote regions in the left hemisphere, and even numbers denote regions in right hemisphere.*

To calculate dynamic FC, we applied a commonly used sliding window approach following our previous study (Yin et al., 2016). Briefly, a tapered window was selected and slid 1 TR, resulting in 168 windows for monkeys and 123 windows for humans. For each time window, Pearson’s correlation coefficients between the mean time courses of any pair of regions were calculated and then a symmetric 82 × 82 connectivity matrix was generated. Thus, dynamic FC matrices were obtained for each participant.

### Mapping Intrinsic Functional Flexibility of Brain

Based on the dynamic FC matrices of each participant, we computed the normalized probability distribution *P_i_ (j…n)* for a given brain region *i* as follows:

$$\begin{array}{c} P_{i\;}(j)\;=\;\frac{n(c_{\mathrm{ij}})}{k\;\times \;w},j=1,2,\ldots ,N,and\;j\neq i \end{array}$$

where *n(c_ij_)* denotes how many times the connection between *i* and *j* emerged across temporal windows, *k* is a predefined threshold indicating number of the strongest connections reserved for region *i* at each time window, and *w* denotes the number of temporal windows. *P_i_(j)* denotes the probability of occurrence for the connection between regions *i* and *j* across all temporal windows. The greater the value of *P_i_(j)*, the more frequent the interaction between region *i* and *j* across the temporal windows, and vice versa.

Regarding the threshold *k*, we have justified the choice of *k* for the human dataset in our previous study (Yin et al., 2016) as follows. Taking into account that brain is organized as a sparse and economical functional network, we first considered a wide range of *k* from 1 to 10. We then calculated the entropy (see definition below) for all brain regions for each *k*. Subsequently, two parameters were calculated, including contrast (identifying the value of k most sensitive to differences in entropy across the whole brain) and consistency (identify the value of *k* where the resulting entropy distribution is most representative of the distributions at other thresholds). We finally summed the two metrics, contrast and consistency, at each threshold *k*, and the peak value of this total was considered as corresponding to the optimal threshold (a peak value emerges at *k* = 3 for human dataset). Considering that the optimal threshold *k* may be different for different states or species, we conducted the above analysis for the anesthetized monkeys in this study. We found that the peak value also emerges at *k* = 3, although the maximum value was at *k* = 1 for the anesthetized monkeys (Supplementary Figure S1). We therefore used the same threshold *k* = 3 for both humans and monkeys.

Subsequently, Shannon entropy *E_i_* was applied to the probability distribution of each brain region *i*:

$$\begin{array}{c} E_{i}=-\sum_{j=1}^{N}P_{i}\left(j\right)\times \log_{2}P_{i}\left(j\right), \end{array}$$

Here, *E_i_* was used to quantify functional flexibility, which characterizes heterogeneous connectivity between region *i* and others over time. A higher value of *E_i_* indicates greater functional flexibility, and vice versa. Readers can see our previous study for details regarding methodology (Yin et al., 2016).

### Mapping Intrinsic Functional Complexity of Brain

The measure of functional complexity based on the distribution of whole-brain correlation values (for static network) without the need for thresholding has been proposed in earlier work (Zhao et al., 2010; Zamora-Lopez et al., 2016). Inspired by this, we calculated complexity *C_i_* for the distribution of correlation values *r_ij_* (for dynamical FC) of a node *i*. Here, we chose to define complexity as the difference between the observed distribution *p(r_ij_)* and the uniform distribution, which is most robust to variations in the number of bins compared with alternatives such as entropy (Zamora-Lopez et al., 2016). The formula is as follows:

$$\begin{array}{c} C_{i}=1-\frac{1}{C_{m}}\sum_{u=1}^{m}\left|p_{u}\left(r_{\mathrm{ij}}\right)-\frac{1}{m}\right|, \end{array}$$

where || means the absolute value, *C_m_* = 2^∗^(*m*-1)/*m*, and m indicates number of bins (here using 50 bins). The *C_i_* reflects functional complexity of a node *i*.

### Mapping Intrinsic Temporal Variability of Brain

A recent study (Zhang et al., 2016) used temporal variability analysis to characterize dynamic functional reconfiguration of each brain region, which can be expressed as follows.

$$\begin{array}{c} \begin{array}{c} V_{i}=1-E\left[corrcoef\left(F_{i,j},F_{i,k}\right)\right],j,k=1,2,3,\ldots w \end{array} \end{array}$$

where *E*[ ] denotes mean value, *F_i,j_* indicates FC profile of node *i* at time window *j* and *w* denotes number of temporal windows. This linear measure does not need a threshold for FC values and is indicative of functional flexibility.

### Time Series Analysis of Brain Activity

To evaluate fluctuations of brain activity, we performed a time series analysis. For the time series of each brain region, we first calculated the distribution of the BOLD signal values with m bins (here using 30 bins). Then, we used entropy to quantify the randomness of fluctuations of brain activity. The higher the entropy of time series, the more random the fluctuations of brain activity. To further test the statistical significance of the randomness, we finally compared real entropy and entropies of 1000 random time series with the same number of time points and number of bins, and a *Z*-score was obtained using the following formula.

$$\begin{array}{c} Z=\frac{H_{\mathrm{real}}-E\left[H_{\mathrm{rand}}\right]}{\mathrm{std}\left(H_{\mathrm{rand}}\right)}, \end{array}$$

where *H_real_* denotes entropy of the observed time series, *H_rand_* denotes entropy of the random time series, and *E*[] indicates mean value. If the *Z*-score approaches zero (theoretically *Z*-score ≤0), the fluctuations of brain activity tend to be random.

### Coupling Between Dynamic and Static Measurements of Spontaneous Brain Activity

To explore the potential effects of anesthesia, we calculated correlations between dynamic measurement (nodal entropy *E*, complexity *C*, and temporal variability *V*) and static measurement (nodal strength, i.e., sum of nodal static FC) within anesthetized monkey and awake human brains, respectively.

### Validation Analysis

To validate our results, we considered the effects of different data processing on interspecies comparisons. Following previous studies (Vincent et al., 2007; Barttfeld et al., 2015; Lv et al., 2016), we used temporal filtering (0.0025–0.05 Hz) for monkeys, which is different from that commonly used for humans (0.01–0.1 Hz). To test the effect of different temporal filtering, we first compared the brain map of entropy *E*, complexity *C*, and temporal variability *V* between different temporal filters in monkeys. We then performed interspecies comparisons using the same temporal filtering (i.e., 0.01–0.1 Hz).

In our main analyses, we used a different number of time points for monkey (*n* = 190) and human (*n* = 145) datasets. To test the effect of a different number of time points, we performed the interspecies comparisons using the same number of time points (*n* = 145).

In the human subject sample, there are 35 subjects with eyes closed and 143 subjects with eyes open. To consider the effect of eye status, we first compared the brain map of entropy *E*, complexity *C*, and temporal variability *V* between the two human subgroups with different eye status. We then performed the interspecies comparisons for both anesthetized monkeys versus human subjects with eyes closed and anesthetized monkeys versus human subjects with eyes open.

## Results

### Static FC Patterns in Anesthetized Monkey and Awake Human Brains

**FIGURE 1:**
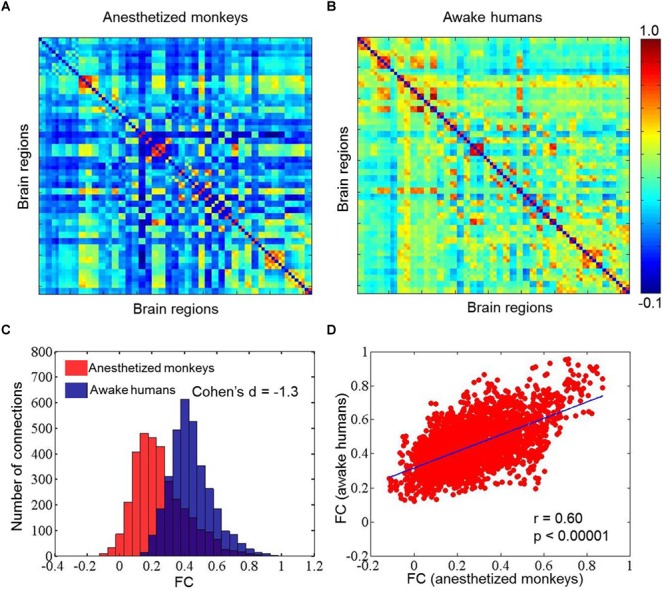
Mean static functional connectivity matrices for anesthetized monkeys **(A)** and awake humans **(B)**; panel **(C)** shows distributions of static functional connectivity for anesthetized monkeys (red) and awake humans (blue); and **(D)** exhibits correlation of static functional connectivity between anesthetized monkeys and awake humans. Color bar denotes Pearson correlation coefficients.

We calculated static, time-averaged FC for both human and monkey datasets. We found that the FC in anesthetized monkeys (mean ±*SD* = 0.25 ± 0.16) was generally weaker than in awake humans (mean ±*SD* = 0.44 ± 0.13) (effect size: Cohen’s *d* = -1.3). However, FC between anesthetized monkeys and awake humans was significantly correlated (*r* = 0.60, *p* < 0.00001) (Figure 1).

### Similarity of Intrinsic Functional Flexibility Between Anesthetized Monkey and Awake Human Brains

**FIGURE 2:**
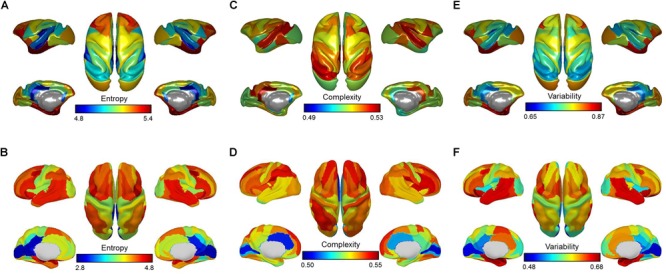
Brain maps of mean entropy for the distribution of dynamic functional connectivity patterns **(A,B)**, complexity for the distribution of correlation values **(C,D)**, and variability for the dynamic functional connectivity patterns **(E,F)** for anesthetized monkeys and awake humans. Color bars denote mean values.

For the anesthetized monkeys, we found that the brain regions with higher entropy *E* mainly involved the lateral prefrontal cortex, anterior insula, and medial temporal lobe. The brain regions that showed lower entropy *E* included primary sensory areas (e.g., auditory and somatosensory regions) and midline default mode regions (e.g., posterior cingulate cortex/retrosplenial cingulate cortex) (Figure 2A).

For the awake humans, we observed that brain regions that showed higher entropy *E* mainly involved the lateral prefrontal, parietal, and temporal cortex, anterior insula, as well as supplementary motor area. The brain regions that showed lower entropy *E* included primary sensory areas (e.g., auditory, visual, and somatosensory regions) and midline default mode regions (e.g., posterior cingulate cortex/retrosplenial cingulate cortex) (Figure 2B). This result is consistent with our previous study (Yin et al., 2016), despite the use of different brain parcellation and dataset.

**FIGURE 3:**
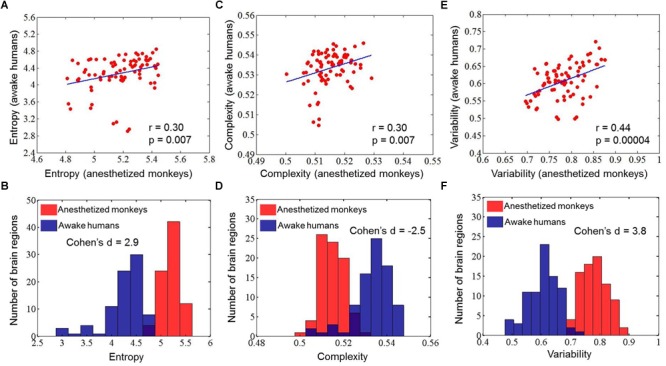
Correlations of mean entropy for the distribution of dynamic functional connectivity patterns **(A)**, complexity for the distribution of correlation values **(C)**, and variability for the dynamic functional connectivity patterns **(E)** between anesthetized monkeys and awake humans. The distribution of mean entropy **(B)**, complexity **(D)**, and variability **(F)** is also shown for anesthetized monkeys and awake humans.

**FIGURE 4:**
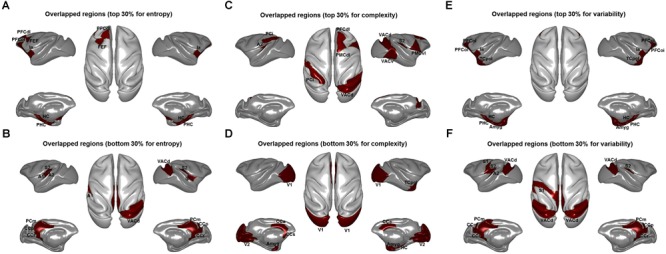
Overlapped brain regions for top 30% entropy values for the distribution of dynamic functional connectivity patterns **(A)**, complexity values for the distribution of correlation values **(C)**, and variability values for the dynamic functional connectivity patterns **(E)** and bottom 30% entropy values for the distribution of dynamic functional connectivity patterns **(B)**, complexity values for the distribution of correlation values **(D)**, and variability values for the dynamic functional connectivity patterns **(F)** between anesthetized monkeys and awake humans. PFCdl, dorsolateral prefrontal cortex; FEF, frontal eye field; PFCol, orbitolateral prefrontal cortex; Ia, anterior insula; HC, hippocampus; PHC, parahippocampal cortex; CCr, retrosplenial cingulate cortex; A1, primary auditory cortex; A2, secondary auditory cortex; VACd, anterior visual area; S2, secondary somatosensory cortex; PCm, medial parietal cortex; Ip, posterior insula; PMCvl, ventrolateral premotor cortex; PCi, inferior parietal cortex; PMCdl, dorsolateral premotor cortex; VACv, anterior visual cortex (ventral part); Amyg, amygdala; V1, primary visual cortex; V2, secondary visual cortex; CCs, subgenual cingulate cortex; TCpol, temporal polar cortex; and S1, primary somatosensory cortex.

Quantitatively, we found a significant correlation of brain-wide entropy *E* between anesthetized monkeys and awake humans (*r* = 0.30, *p* = 0.007) (Figure 3A), although averaged entropy *E* across the whole brain of anesthetized monkeys (mean ±*SD* = 5.18 ± 0.18) was higher than that of awake humans (mean ±*SD* = 4.27 ± 0.41) (effect size: Cohen’s *d* = 2.9) (Figure 3B). These findings indicate that the heterogeneous flexibility across brain regions is preserved in anesthetized monkeys. However, there are some inconsistencies. For example, the primary visual cortex showed relatively higher entropy *E* in the anesthetized monkeys, and relatively lower entropy *E* in the awake humans. In contrast, the inferior parietal cortex exhibited relatively lower entropy *E* in the anesthetized monkeys, but higher in the awake humans. By comparing brain regions with top 30% and bottom 30% entropy *E* values in anesthetized monkeys and awake humans, we found that overlapping regions with higher entropy *E* between species included the left dorsolateral prefrontal cortex, left frontal eye field, left orbitolateral prefrontal cortex, bilateral anterior insula, bilateral orbital inferior prefrontal cortex, bilateral hippocampus, and bilateral parahippocampal cortex (Figure 4A); and that overlapping regions with lower entropy *E* between species included the bilateral posterior cingulate cortex, bilateral retrosplenial cingulate cortex, left primary auditory cortex, left secondary auditory cortex, right anterior visual area, bilateral secondary somatosensory cortex, bilateral medial parietal cortex, and right posterior insula (Figure 4B).

### Comparison of Functional Complexity Measurement With Our Method

**FIGURE 5:**
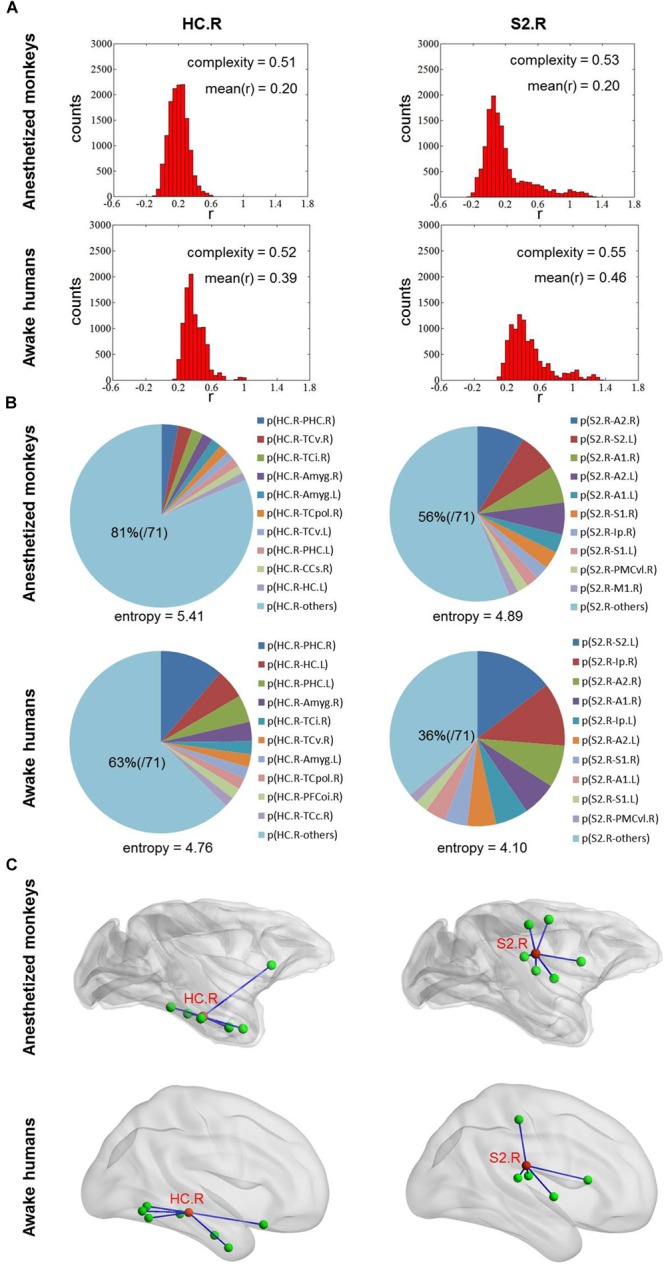
**(A)** Shows the distribution of correlation values for right HC and S2 in both anesthetized monkeys and awake humans. The distribution of correlation values of right HC is narrower (lower complexity) than that of right S2 in both monkeys and humans. **(B)** Shows the probability distribution of strongest connectivity with right HC and S2 in monkeys and humans. The distribution of strongest connectivity with right HC is more uniform (higher flexibility) across brain than that of right S2 in both monkeys and humans. The most frequent connections with right HC and S2 are rendered in **C**. The patterns of most frequent connections are similar between species for both right HC and S2. HC, hippocampal cortex and S2, secondary somatosensory cortex.

We found that overall complexity *C* was higher in awake humans compared with anesthetized monkeys, and the correlation of brain-wide complexity *C* between anesthetized monkeys and awake humans was similar with that obtained using our method (Figure 2C,D, 3C,D). However, few of the well-known flexible cognitive control regions such as lateral prefrontal cortex, anterior insula, and hippocampal cortex showed higher complexity *C* in anesthetized monkeys (Figure 4C). Instead, we observed unimodal regions such as secondary somatosensory cortex, auditory cortex, and visual cortex showed higher complexity *C* (Figure 4D). It is possible that this complexity measurement *C* based on the distribution of correlation values is not suitable for quantifying the heterogeneous functional flexibility of the brain, although it can better assess the functional complexity of the system at different states. For instance, we found the hippocampal cortex exhibited narrower distribution of correlation values (lower complexity) than that of secondary somatosensory cortex in both anesthetized monkeys and awake humans, whereas the time-varying strongest connections of hippocampal cortex was more uniform (higher flexibility) across the whole brain than that of secondary somatosensory cortex (Figure 5).

**FIGURE 6:**
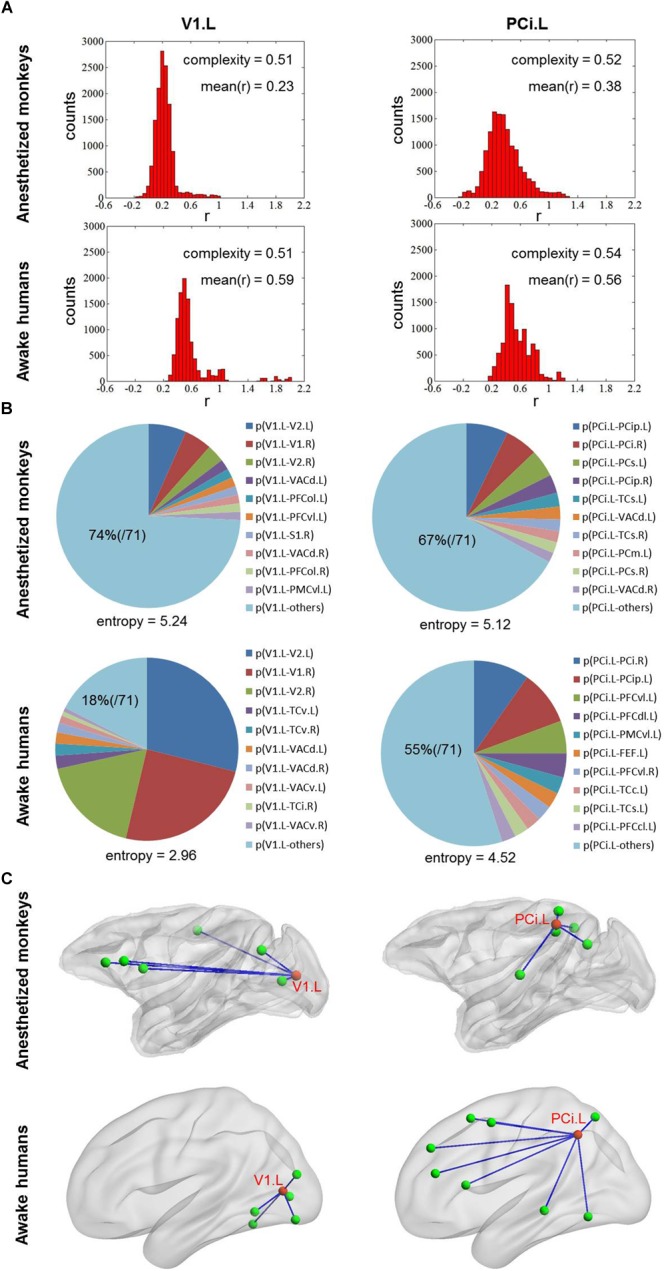
**(A)** Shows the distribution of correlation values for left V1 and PCi in both anesthetized monkeys and awake humans. The distribution of correlation values of left V1 is narrower (lower complexity) than that of left PCi in both monkeys and humans. **(B)** Shows probability distribution of strongest connectivity with left V1 and PCi in monkeys and humans. The distribution of strongest connectivity with left PCi is more uniform (higher flexibility) across brain than that of left V1 in humans, which the opposite is seen in monkeys. The most frequent connections with left V1 and PCi are rendered in **C**. The patterns of most frequent connections are different between species for both left V1 and PCi. V1, primary visual cortex and PCi, inferior parietal cortex.

Additionally, we found right inferior parietal cortex showed higher complexity *C* and primary visual cortex showed lower complexity *C* in both anesthetized monkeys and awake humans (Figure 4C,D). However, we observed that the strongest connections of primary visual cortex were local and stereotyped in awake humans while distributed and variable across brain in anesthetized monkeys. Moreover, we found inferior parietal cortex more frequently connected with local brain regions in parietal, temporal, and visual cortices in monkeys while more frequently connected with broad brain regions in parietal, temporal, and frontal cortices in humans (Figure 6).

### Comparison of Time Variability Measurement With Our Method

We found the results obtained by temporal variability measurement *V* were highly consistent with that using our method, but not complexity measurement *C* based on distribution of correlation values (Figure 2E,F, 3E,F, 4E,F). This suggests that information of spatial connectivity patterns is more important than distribution of correlation values for describing functional flexibility, even with *k* (= 3) strongest connections at each time window.

### Time Series Analysis of Brain Activity

**FIGURE 7:**
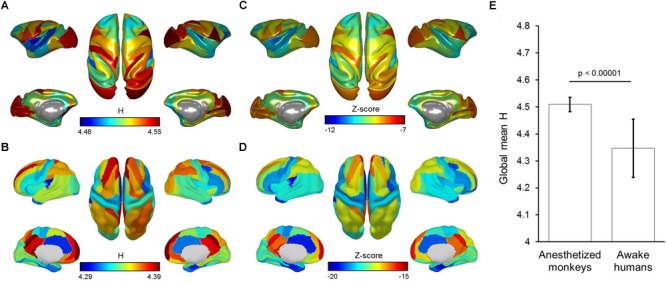
Brain map of mean entropy of time series for anesthetized monkeys **(A)** and awake humans **(B)**. Brain map of mean *Z*-scores for entropy of time series are also shown for monkeys **(C)** and humans **(D)**. **(E)** Shows global mean entropy of time series for monkeys and humans. Color bars denote mean values.

We found the entropy *H* of time series to be globally higher in anesthetized monkeys compared with awake humans. In particular, we observed that the primary visual cortex showed the highest entropy *H* in anesthetized monkeys. However, entropy *H* of time series for all brain regions were remarkably lower than that of random time series in both anesthetized monkeys and awake humans (Figure 7). This result suggests that brain activity in anesthetized monkeys generally shifts toward the random fluctuations, but still differs from random fluctuations. In addition, the effect of anesthesia on fluctuations of brain activity is probably non-uniform across brain.

### Distinct Relationships Between Dynamic and Static Measurements Within Awake Human and Anesthetized Monkey Brains

**FIGURE 8:**
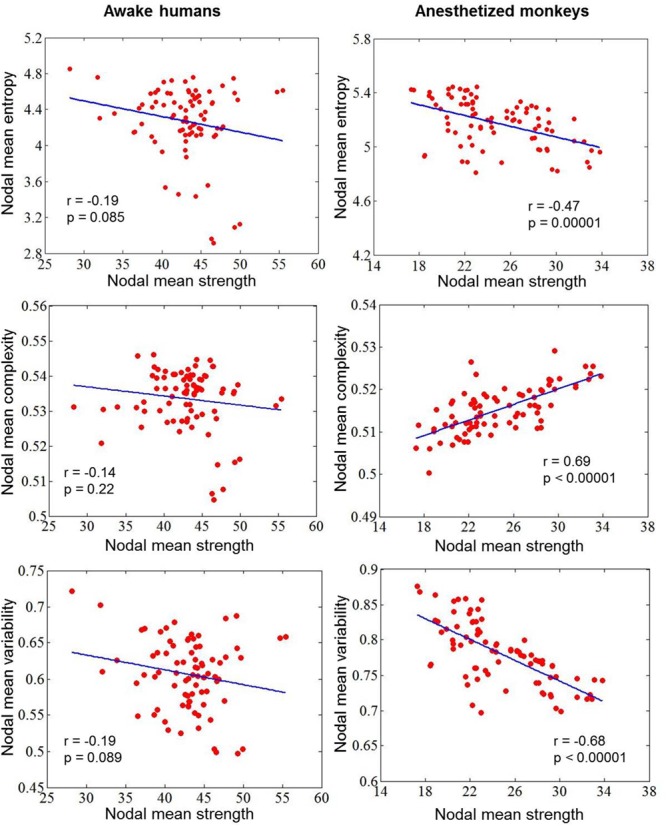
Correlations between dynamic measurement (nodal mean entropy for the distribution of dynamic functional connectivity patterns, complexity for the distribution of correlation values, and variability for the dynamic functional connectivity patterns) and static measurement (nodal mean FC strength) in awake humans and anesthetized monkeys.

To explore the impacts of anesthesia, we calculated correlations between dynamic measurement and static measurement in awake humans and anesthetized monkeys, respectively. We found a negative correlation (*r* = -0.47, *p* = 0.00001) between nodal entropy *E* and strength in anesthetized monkeys, but not in awake humans (*r* = -0.19, *p* = 0.085). In contrast, we found a positive correlation (*r* = 0.69, *p* < 0.00001) between nodal complexity *C* and strength in anesthetized monkeys, but not in awake humans (*r* = -0.14, *p* = 0.22). Consistent with our method, we found a negative correlation (*r* = -0.68, *p* < 0.00001) between nodal temporal variability *V* and strength in anesthetized monkeys, but not in awake humans (*r* = -0.19, *p* = 0.089) (Figure 8).

### Validation Analysis

Regarding the effect of temporal filtering, we found significant correlations between two different temporal filters in monkeys for entropy *E* (*r* = 0.97, *p* < 0.00001), complexity *C* (*r* = 0.73, *p* < 0.00001), and variability *V* (*r* = 0.96, *p* < 0.00001) (Supplementary Figure S2). Moreover, we consistently observed significant correlations between species for entropy *E* (*r* = 0.26, *p* = 0.02), complexity *C* (*r* = 0.30, *p* = 0.007), and variability *V* (*r* = 0.37, *p* = 0.0007) with the same time filtering (Supplementary Figure S3). However, the interspecies correlations based on the same temporal filtering were a little weaker than when using different temporal filtering for entropy *E* and variability *V*, but the same for complexity *C*. This result indicates that different temporal filtering for monkeys and humans may give better correspondence between species.

For the effect of different number of time points, we consistently observed significant correlations between species for entropy *E* (*r* = 0.28, *p* = 0.01), complexity *C* (*r* = 0.30, *p* = 0.005), and variability *V* (*r* = 0.44, *p* = 0.00003) with the same number of time points (Supplementary Figure S4). This result suggests that the different number of time points used for monkeys and humans did not significantly affect correlations between species.

For the effect of eye status, we found significant correlations between the two human subgroups for entropy *E* (*r* = 0.96, *p* < 0.00001), complexity *C* (*r* = 0.75, *p* < 0.00001), and variability *V* (*r* = 0.91, *p* < 0.00001) with different eye status (Supplementary Figure S5). For the interspecies comparisons, we found the correlations were similar for the two conditions of human subjects with different eye status (Supplementary Figure S6). This result suggests that our main findings are not significantly affected by eye status of human subjects during resting-state fMRI scanning. Specifically, we observed that the visual cortex showed lowest entropy *E* in the human subjects for both closed and open eyes. This further implies that eye status does not change the rank of functional flexibility of visual cortex in the brain. In the anesthetized monkeys, the primary visual cortex showed relatively high entropy *E*. This is probably because anesthesia causes brain activity in primary visual cortex to shift much more toward random fluctuations.

## Discussion

Although resting-state connectivity networks have been used for decades to probe functional brain organization (Deco et al., 2011; Raichle, 2015; Bassett and Sporns, 2017), the origins and functional significance of resting-state connectivity patterns require further understanding. In a recent study, we were able to characterize the heterogeneous architecture of intrinsic functional flexibility in the awake, resting human brain using dynamic FC analysis and a probabilistic model (Yin et al., 2016). To further understand functional organization of spontaneous brain activity, in this study, we performed an interspecies comparison of intrinsic functional flexibility between anesthetized monkeys and awake humans.

For reference, we compared conventional, static FC patterns between anesthetized monkeys and awake humans. We found that functional coupling between brain regions in anesthetized monkeys was generally weaker than in awake humans; however, brain-wide connectivity patterns were correlated between species. Previous studies showed that patterns of static FC, such as the default mode network, persist even after loss of consciousness in rodents (Liang et al., 2012; Lu et al., 2012) and primates (Vincent et al., 2007; Hutchison et al., 2011; Hutchison and Everling, 2012). In contrast, other studies suggested a breakdown of both within- and between-network resting-state connectivity in the anesthetized state (Boveroux et al., 2010; Stamatakis et al., 2010) and deep sleep state (Tagliazucchi et al., 2013). These studies raised two hypotheses about the origins of resting-state FC patterns: reflecting a continuous stream of ongoing cognitive process and random fluctuations constrained by a stable anatomical skeleton (Vincent et al., 2007; Honey et al., 2009; Barttfeld et al., 2015; Raichle, 2015). It is possible that persisting static FC patterns during anesthesia can be attributed to anatomical constraints (Vincent et al., 2007; Deco et al., 2013). On the other hand, consciousness is probably indexed by global integration with strong couplings between long-range brain regions (Alkire et al., 2008).

For our dynamic analysis, we found that the brain regions that showed higher entropy *E* in both anesthetized monkeys and awake humans mainly involved the higher-order association cortex such as the lateral prefrontal cortex, and regions that showed lower entropy *E* included primary sensory areas and midline default mode regions. This result is consistent with the flexible hub theory: the FC patterns of frontoparietal regions shift more than those of other regions across a variety of task states (Cole et al., 2013). A previous study focusing on the temporal dynamics of resting-state functional networks suggested that wakefulness is characterized by the dynamical exploration of a richer repertoire of functional configurations or states (Barttfeld et al., 2015). Moreover, individuals with brain networks showing greater dynamics perform more favorably in behavioral tasks (Jia et al., 2014). Through comparing anesthetized monkeys with awake humans, Hutchison and his colleagues demonstrated that the temporal dynamics of resting-state FC are also an intrinsic property of brain organization and not simply a consequence of conscious or cognitive processing (Hutchison et al., 2013b). Moreover, there is accumulating evidence that many cognitive processes can occur in the absence of awareness (MacDonald et al., 2015). We have demonstrated that brain regions showing higher entropy *E* may represent a more flexible exploration of functional configurations, even under anesthesia. Expanding upon previous studies (Cole et al., 2013; Yin et al., 2016; Zhang et al., 2016), this study reveals that heterogeneous functional flexibility across the cortex is evolutionarily conserved and persists across brain states.

Although there is significant correlation of brain-wide entropy *E* between anesthetized monkeys and awake humans, the whole-brain average entropy *E* is remarkably higher in the anesthetized monkeys. Barttfeld et al. study indicated that anesthesia may lead to a stable brain state that is more similar to the structure, in which time series of brain activity resemble random fluctuations shaped by fixed anatomical connectivity (Barttfeld et al., 2015). Using dynamical systems modeling, a previous study further suggested that low coupling strength between brain regions can coexist with a single stable spontaneous connectivity pattern (Deco et al., 2013). Because it becomes the only available attractor, the sedated brain cannot depart from it and remains confined to a semirandom exploration of the valley surrounding it, thus simultaneously exhibiting interregional correlations along with fixed anatomical connectivity and a memoryless trajectory (Deco et al., 2013; Barttfeld et al., 2015). Consistently, through time series analysis, we found that brain activity in anesthetized monkeys generally shifted toward random fluctuations, but it was still different from random fluctuations. We speculate that the general increase in entropy *E* and decrease in static FC strength in the anesthetized monkey brain is likely attributable to anesthesia-induced random fluctuations of brain activity.

Notably, previous electrophysiological studies have suggested that brain neuronal activity is dominant with slow oscillations under anesthetic states as well as during deep sleep state (Isomura et al., 2006; Alkire et al., 2008; Ni Mhuircheartaigh et al., 2013). A remarkable feature of slow oscillation is the synchrony over large cortical areas (Achermann and Borbely, 1997; Destexhe et al., 1999). Breshears et al. (2010) have demonstrated that stable functional architecture and dynamic neural activity are concurrent during induction of anesthesia. It is possible that the stable functional architecture, i.e., large-scale functional networks frequently observed even in anesthesia (Vincent et al., 2007; Hutchison and Everling, 2012), is result from synchrony induced by slow oscillation. Regarding dynamics of brain activity, a breakdown of long-range temporal correlations was observed in BOLD signals during both anesthesia and deep sleep states, suggesting that the dynamics of time series is close to white noise (Tagliazucchi et al., 2013; Barttfeld et al., 2015). In despite of different temporal scales, the findings that BOLD signal appears to be more random can provide a supplement for understanding anesthesia-induced slow oscillations of electrophysiological activity.

Moreover, previous evidence suggests that the dynamic complexity of the system under anesthetized state is reduced (Barttfeld et al., 2015). Accordingly, we found an overall reduction of functional complexity in anesthetized monkeys based on a complexity measure *C* for distribution of correlation values, but this was not the case for our method and time variability measurement. This suggests that the complexity measure *C* for distribution of correlation values is better to quantify dynamic complexity of system. In contrast, our method and time variability measurement are more suitable for describing functional flexibility, because they enable the capture of information regarding dynamical spatial connectivity patterns of a node, while this is not the case for the complexity measurement based on distribution of correlation values. For instance, the hippocampal cortex exhibited narrower distribution of correlation values (lower complexity) than the secondary somatosensory cortex in both anesthetized monkeys and awake humans, whereas the time-varying strongest connections of hippocampal cortex were more uniform (higher flexibility) than that of secondary somatosensory cortex across the whole brain. Our findings suggest that combining different methods could provide more complete information for in-depth understanding of functional brain organization.

We further found a negative correlation between nodal entropy *E* and strength in anesthetized monkeys, but not in awake humans. Previous simulating and empirical data suggest that brain dynamics may change from a single stable state to multi-stable state, as coupling strength between brain areas increases from the sedated to the conscious condition (Dehaene and Changeux, 2005; Ghosh et al., 2008; Deco et al., 2011, 2013; Hudetz et al., 2015). In agreement, conscious processing is supported by global integration with strong coupling between long-distance brain regions as well as a diversity of cognitive states (Alkire et al., 2008; Dehaene and Changeux, 2011). Loss of consciousness due to anesthesia may lack both strong coupling and a rich repertoire of cognitive states (Barttfeld et al., 2015; Hudetz et al., 2015). A possible explanation is that the negative correlation between nodal entropy *E* and strength (i.e., the weaker connectivity strength, the higher entropy *E*) is primarily dominated by random fluctuations of brain activity induced by anesthesia. In contrast, the wakeful condition or conscious access with dominance of heterogeneous cognitive states may lead to decoupling between nodal entropy *E* and strength.

Consistently, we observed no correlation between the complexity measurement *C* for distribution of correlation values and static connectivity strength for the awake humans, but there was a positive correlation for the anesthetized monkeys. Zamora-Lopez et al. (2016) reported a reverse U-shaped relationship between functional complexity and coupling strength, with an optimal functional organization at the peak complexity. In other words, the functional complexity increases and then decreases during the increase of coupling strength. We therefore speculate that positive correlation between the complexity measurement *C* and static connectivity strength in anesthetized monkeys is attributed to the anesthesia-induced lower coupling strength (at the left part of reverse U-shape). This result further suggests that the coupling between dynamic and static measures may serve as a potential signature of anesthesia, whereas the direction of correlation is probably dependent on specific metrics.

Although brain-wide correlation exists between species, it should be noted that there were some divergences in intrinsic functional flexibility between the anesthetized monkey and awake human brains. For instance, the primary visual cortex exhibited relatively high entropy *E* in the anesthetized monkeys, although it was low in the awake humans. In contrast, the inferior parietal cortex showed relatively low entropy *E* in the anesthetized monkeys, whereas it was high in the awake humans. A previous human study indicated that anesthesia preferentially modulates higher-order connections, but not low-level sensory connections (Martuzzi et al., 2010). One rodent study also showed that anesthesia profoundly impacted the dynamic resting-state FC of neural circuits subserving higher-order functions but had less effect on sensory systems (Liang et al., 2015). On the other hand, a study by Hudetz et al. (2015) reported that the largest reduction of temporal variance of BOLD signals occurred in the visual cortex and parietal cortex in anesthetized rats. Although conflicting conclusions were drawn in previous studies, the converging evidence suggests a non-uniform impact of anesthesia on brain systems. In anesthetized monkeys, we found brain activity of primary visual cortex and sensorimotor cortex to be much more close to random fluctuations. In addition, the strongest connections of primary visual cortex were local and stereotyped in awake humans, but distributed and variable across the brain in anesthetized monkeys. It is possible the much more random fluctuations may contribute to the difference of functional flexibility observed in primary visual cortex between species.

Regarding inferior parietal cortex, we found it to be more frequently connected with local brain regions in parietal, temporal, and visual cortices in monkeys while more frequently connected with broad brain regions in parietal, temporal, and frontal cortices in humans. From an evolutionary perspective, the inferior parietal cortex in the human brain mainly contains Brodmann areas 39 and 40, but monkeys do not have a comparable area (Kotter and Wanke, 2005; Raichle, 2015). We speculate that the difference of functional flexibility observed in inferior parietal cortex of monkeys and humans is likely attributed to evolution. Although evolution may indeed result in functional reorganization of specific brain regions, it is hard to separate the contributions of evolution and anesthesia in the current study. A further study with awake monkeys and anesthetized humans may help clarify this question.

In addition, there are some limitations to this study. First, our analysis is based on dynamic FC and is affected by the general limits of this technique, such as temporal resolution of fMRI (Hutchison et al., 2013a; Hindriks et al., 2016). Using simultaneous imaging and electrophysiological recording is helpful for interpretation of dynamic FC (Keilholz, 2014). Second, for a direct comparison of brain network between monkeys and humans, we used a regional map template with the same cortical partitions. Previous studies have suggested evolutionary differences in anatomy between monkeys and humans (Kotter and Wanke, 2005; Raichle, 2015). Our findings may be potentially affected by the anatomical differences resulting from primate evolution. Finally, the human data that was collected from multiple centers with different acquisition parameters likely contained non-trivial variability across sites and individuals.

## Summary

This study combined dynamical complexity measurements and static connectivity strength measurement to understand functional brain organization in anesthetized monkeys and awake humans. Cross-species comparison suggests that the heterogeneous brain map of intrinsic functional flexibility persists during primate evolution and transcends levels of consciousness, remaining present under anesthesia. Moreover, the coupling between dynamic and static measurements can provide a potential signature of loss of consciousness due to anesthesia. However, each method may capture different biological information and have its own limitation. Specifically, our method and temporal variability approach might be more suitable for describing functional flexibility of a node, whereas there is a potential flaw in characterizing the changes of system complexity induced by anesthesia. In contrast, the complexity measurement based on distribution of correlation values is better for evaluating functional complexity of systems with different states or coupling strength, but is probably not suitable for describing functional flexibility of a node due to failure in capturing information of spatial connectivity patterns. Combining different methods could provide more complete information for in-depth understanding of functional brain organization. This study not only offers fresh insight into evolution of functional brain organization, but also advances our understanding of dynamics of spontaneous brain activity.

## Data Availability

The datasets generated for this study are available on request to the corresponding author.

## Author Contributions

DY, YW, and ZheW designed the research. DY, ZZ, and ZhiW performed the research. DY, ZhiW, QL, and DC analyzed the data. DY, KZ, and ZheW wrote the paper.

## Conflict of Interest Statement

The authors declare that the research was conducted in the absence of any commercial or financial relationships that could be construed as a potential conflict of interest.

## Footnotes

1 http://fcon_1000.projects.nitrc.org/indi/abide/

2 http://www.fil.ion.ucl.ac.uk/spm

3 http://www.fmrib.ox.ac.uk

4 http://sumsdb.wustl.edu/sums/macaquemore.do

5 http://preprocessed-connectomes-project.org/abide/index.html

6 http://preprocessed-connectomes-project.org/abide/dparsf.html
