# Supplementary figures and images for: Brain Map of Intrinsic Functional Flexibility in Anesthetized Monkeys and Awake Humans
Source: Front Neurosci. 2019 Feb 28;13:174. doi: 10.3389/fnins.2019.00174 (PMC6403192; doi:10.3389/fnins.2019.00174)

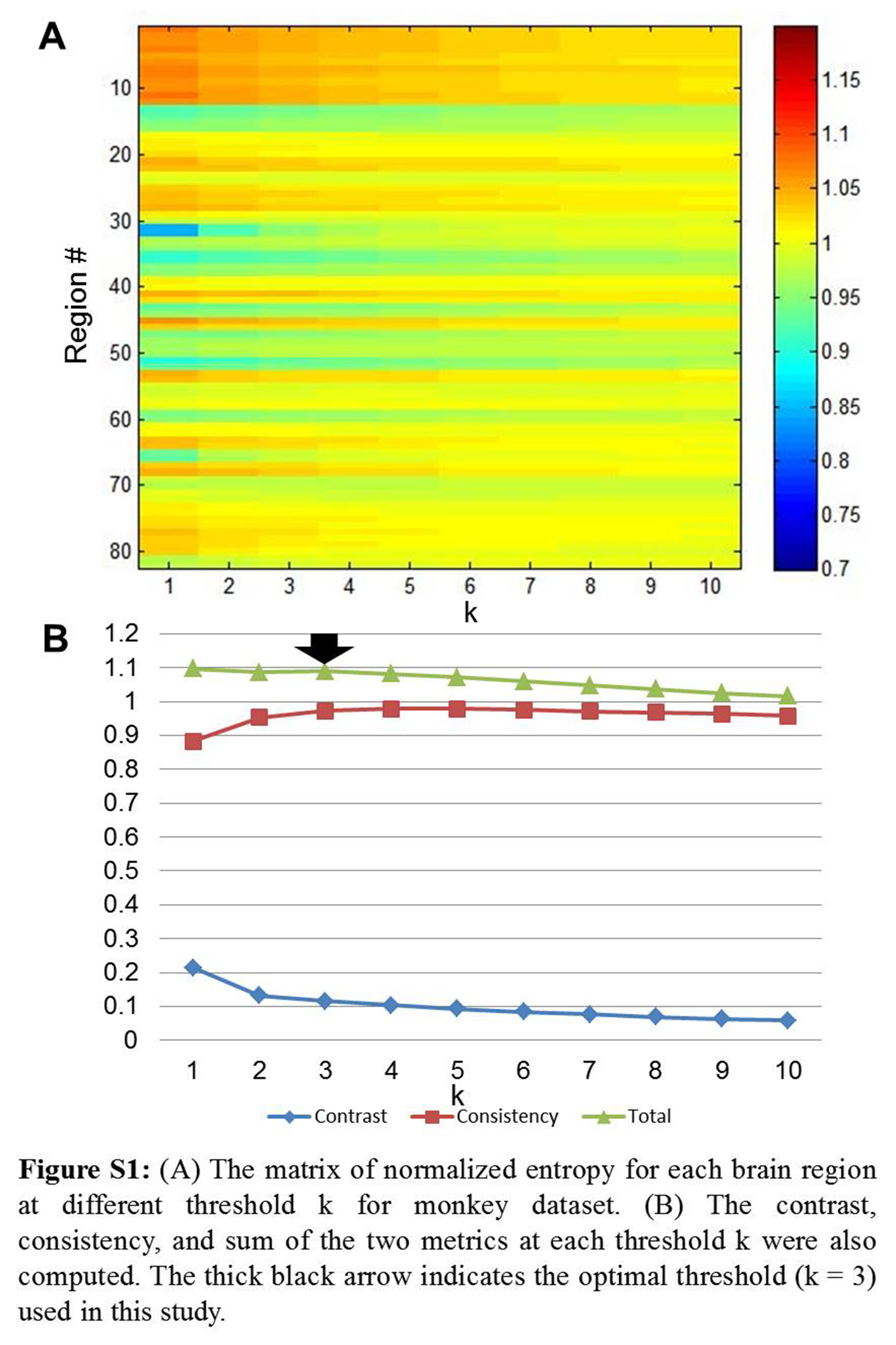

Supplement: Supplementary file 1 [file Image_1.TIF]

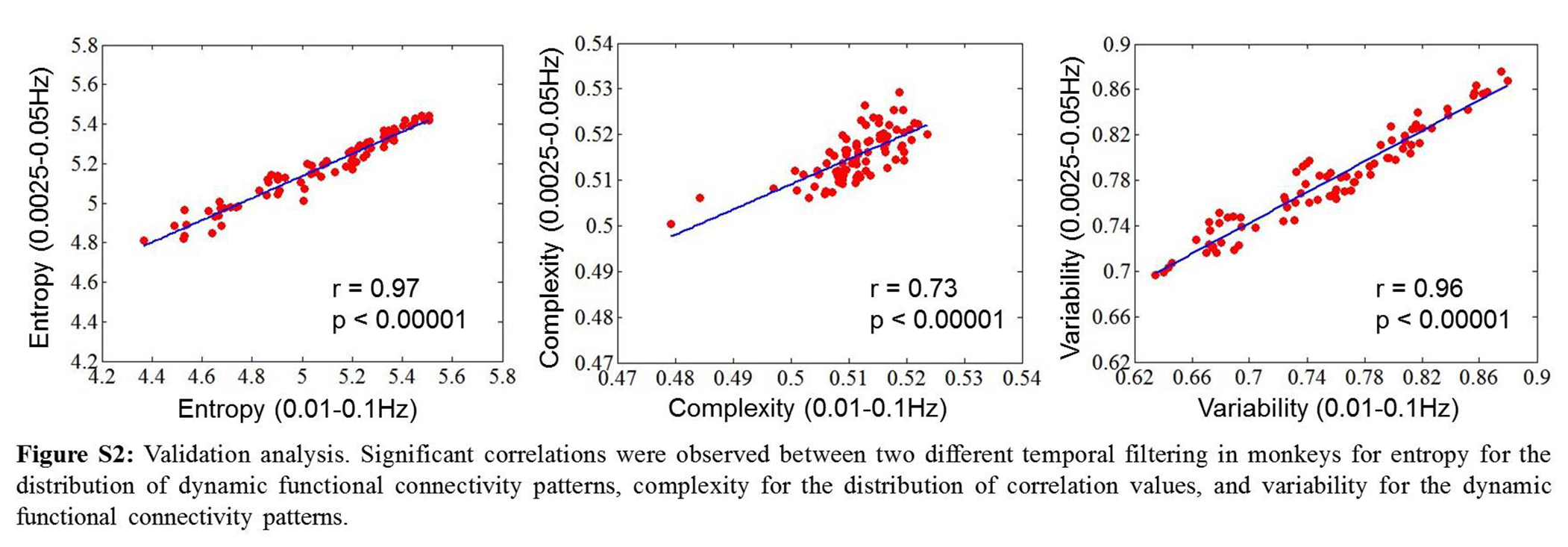

Supplement: Supplementary file 2 [file Image_2.TIF]

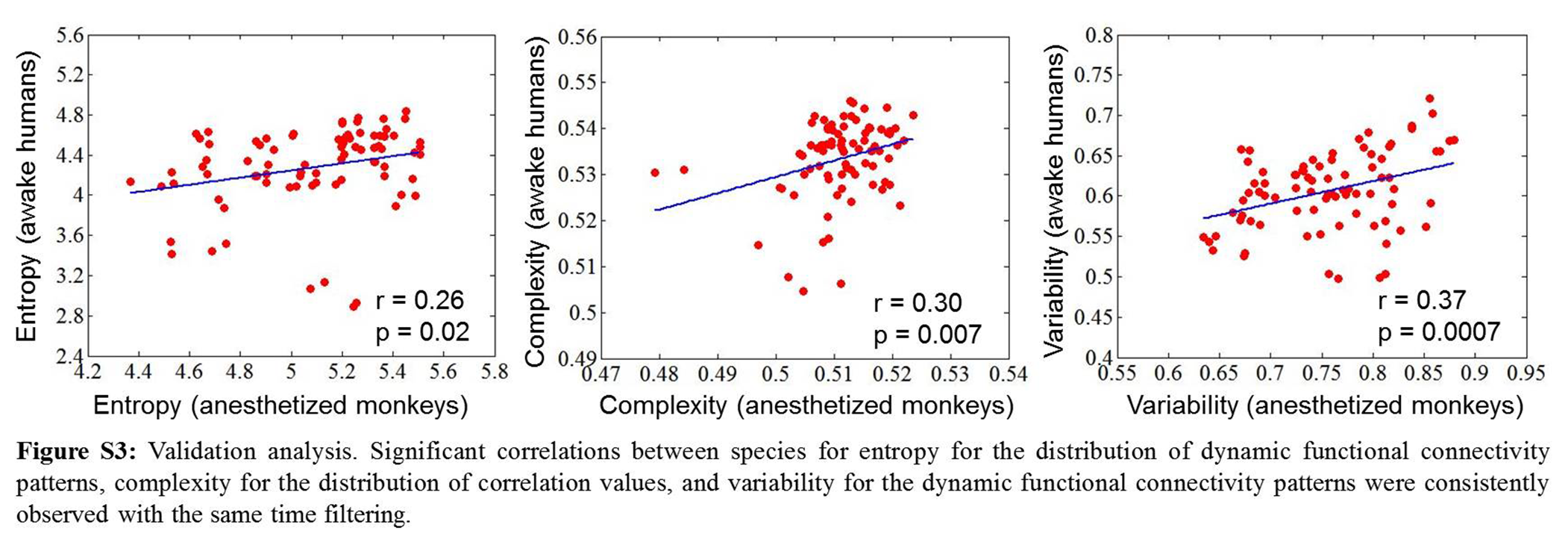

Supplement: Supplementary file 3 [file Image_3.TIF]

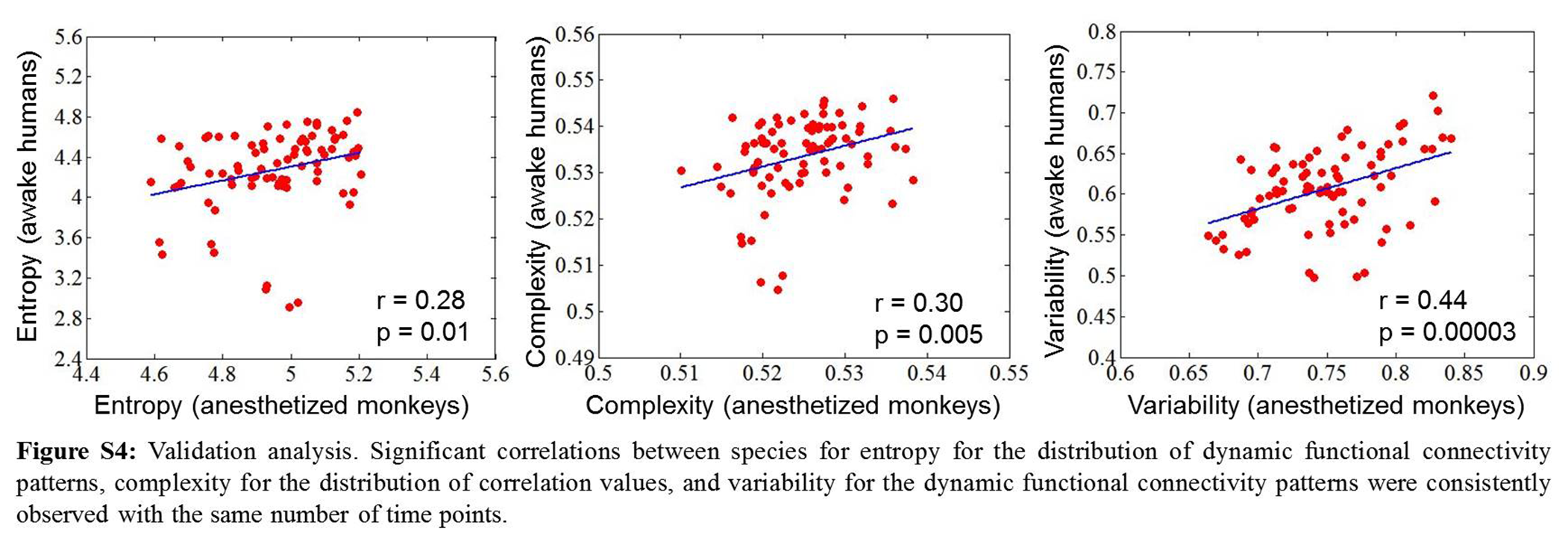

Supplement: Supplementary file 4 [file Image_4.TIF]

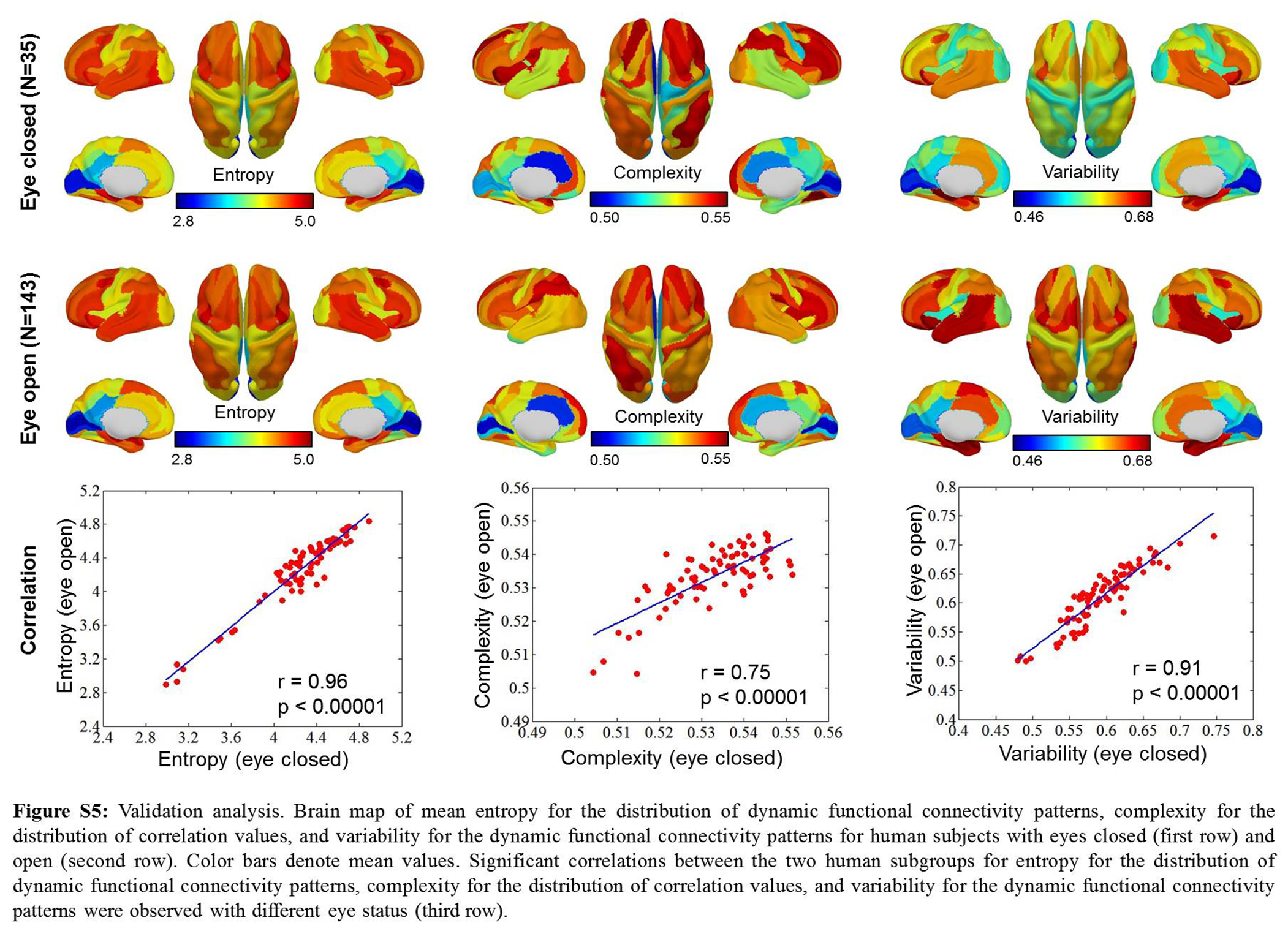

Supplement: Supplementary file 5 [file Image_5.TIF]

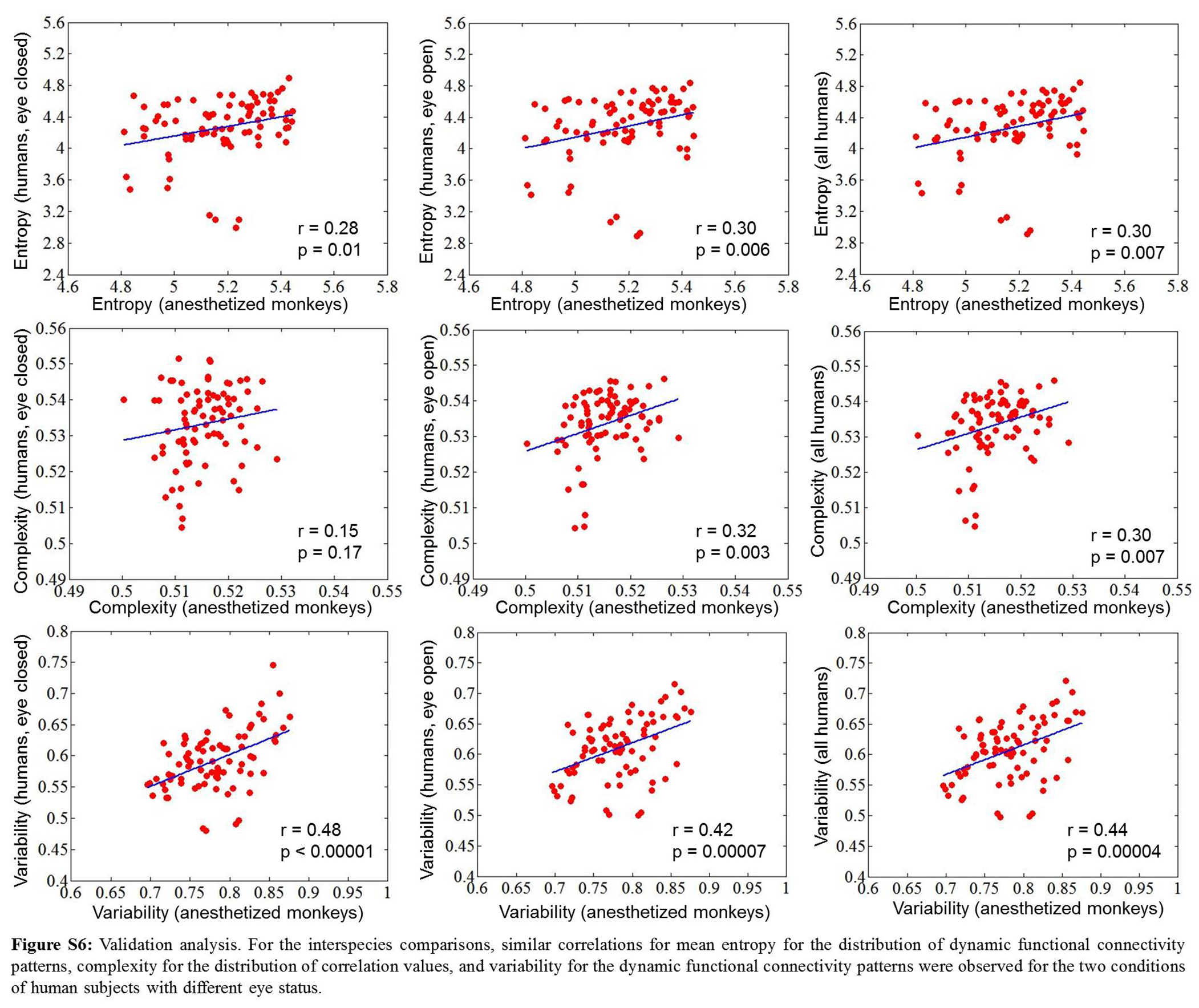

Supplement: Supplementary file 6 [file Image_6.TIF]
